# A Robust Method to Analyze Copy Number Alterations of Less than 100 kb in Single Cells Using Oligonucleotide Array CGH

**DOI:** 10.1371/journal.pone.0067031

**Published:** 2013-06-25

**Authors:** Birte Möhlendick, Christoph Bartenhagen, Bianca Behrens, Ellen Honisch, Katharina Raba, Wolfram T. Knoefel, Nikolas H. Stoecklein

**Affiliations:** 1 Department of Surgery (A), Heinrich-Heine University and University Hospital Düsseldorf, Düsseldorf, Germany; 2 Institute of Medical Informatics, University of Münster, Münster, Germany; 3 Department of Obstetrics and Gynecology, Heinrich-Heine University and University Hospital Düsseldorf, Düsseldorf, Germany; 4 Institute for Transplantation Diagnostics and Cell Therapeutics, Heinrich-Heine University and University Hospital Düsseldorf, Düsseldorf, Germany; University of Navarra, Spain

## Abstract

Comprehensive genome wide analyses of single cells became increasingly important in cancer research, but remain to be a technically challenging task. Here, we provide a protocol for array comparative genomic hybridization (aCGH) of single cells. The protocol is based on an established adapter-linker PCR (WGAM) and allowed us to detect copy number alterations as small as 56 kb in single cells. In addition we report on factors influencing the success of single cell aCGH downstream of the amplification method, including the characteristics of the reference DNA, the labeling technique, the amount of input DNA, reamplification, the aCGH resolution, and data analysis. In comparison with two other commercially available non-linear single cell amplification methods, WGAM showed a very good performance in aCGH experiments. Finally, we demonstrate that cancer cells that were processed and identified by the CellSearch® System and that were subsequently isolated from the CellSearch® cartridge as single cells by fluorescence activated cell sorting (FACS) could be successfully analyzed using our WGAM-aCGH protocol. We believe that even in the era of next-generation sequencing, our single cell aCGH protocol will be a useful and (cost-) effective approach to study copy number alterations in single cells at resolution comparable to those reported currently for single cell digital karyotyping based on next generation sequencing data.

## Introduction

Genetic instability, clonal selection and evolution seem to be important driving forces of cancer progression. The resulting genetic heterogeneity is a hallmark of cancer [1]–[3]. Although genetic heterogeneity of cancer is well-known, comprehensive and systematic analyses of this phenomenon are quite rare; especially studies comprising a more complete spectrum of the disease, e.g. primary tumors and matched disseminated cancer cells, micrometastases and/or metastasis [2]. Recent studies on multiple tumor biopsies and subsequent next-generation sequencing approaches [4], [5] revealed a surprisingly high degree of heterogeneity within individual cancers. However, understanding the full extent of genetic heterogeneity between cancer cells ultimately requires analyses on single cell level. A precise assessment is not only important from a tumor-biologic point of view but becomes vital in the era of molecular therapy, because it likely contributes significantly to therapy resistance [6]–[9]. In addition, cancer cell populations relevant for disease progression can be quite rare and are only assessable as small cell populations or even single cells, e.g. tumor initiating/tumor stem cells, disseminating (DTCs) or circulating tumor cells (CTCs). Therefore, robust single cell profiling protocols are needed for comprehensive interrogation of genomic alterations in single cancer cells.

More than a decade ago, Klein *et al.* described an adapter-linker PCR (AL-PCR) approach for whole genome amplification (WGAM, [10]) of single immuno-detected DTCs and subsequent genomic analysis by gene sequencing and metaphase-based comparative genomic hybridization (mCGH). This allowed a more detailed genetic characterization of single DTCs for the first time providing new important insights into systemic cancer progression [11], which were also of significant clinical relevance [11], [12]. The main method to assess genome wide copy number alterations (CNAs) in those studies [13]–[16] was mCGH. This method proved to be very reliable and robust for the hybridization of single cell amplification products. Clearly, mCGH has several inherent limitations, including a low resolution of only 5–10 Mb and a very laborious, time-consuming protocol that is difficult to standardize. For genomic DNA mCGH is rather outdated and replaced by oligonucleotide microarray CGH (aCGH) and more recently by digital karyotyping using next-generation sequencing approaches [17]–[19]. In order to further improve single cell genomic profiling using WGAM, the compatibility of amplification products with different array CGH platforms has been tested [20]. Only a specifically designed BAC-array using pulse field gel electrophoresis (PFGE) purified DNA was a reliable array CGH method providing a resolution down to 1 Mb. At that time oligonucleotide aCGH was found to be clearly inferior to the PFGE-BAC array and was considered as incompatible with WGAM. However, after revisiting the originally used protocol we made changes to the method that finally enabled successful aCGH using the 4×180k Agilent Technologies platform.

The concept of this study was to evaluate the robustness of our aCGH protocol for elucidating genome wide CNAs in single cell WGAM products. We chose Agilent’s SurePrint G3 arrays since (i) it was shown as the currently best performing aCGH platform [21], (ii) its wide availability and (iii) because of its cost-effectiveness per experiment. We also report on requirements and various optimizations of the basic WGAM-based aCGH protocol regarding reamplification, labeling and data analysis. Finally, we investigated two other commercially available non-linear PCR-based whole genome amplification methods for single cells to compare their performance in aCGH experiments.

## Materials and Methods

### Preparation of Single Cells

Mononuclear cells were prepared from peripheral blood (PBMNCs) of a female (BM) as well as a male (NHS) healthy control using Ficoll gradient centrifugation. For single cell isolation of mononuclear cells the bottom of a petri dish was coated with FCS, cell suspensions (healthy controls, cell lines REH and OE19) were diluted with 1× PBS to achieve a density of one cell per visual field under an inverse microscope with 100× magnification. Culture conditions of the used cell lines and genomic DNA preparation are given in methods S1 and S2. Single cells were picked under visual control using a 1 µl pipette and transferred directly into the respective buffer for subsequent whole genome amplification methods.

### Ethics Statement

For the voluntary donation of blood for a female healthy control by the author BM and a male healthy control by the author NHS for the preparation of gDNA and PBMNCs as a reference in the aCGH analyses the ethical approval was obtained by the ethics committee of the medical faculty of the Heinrich-Heine University Düsseldorf. Because the voluntary donation of the author’s blood was in their own interest a written consent was waived, which was approved by the ethics committee of the medical faculty of the Heinrich-Heine University Düsseldorf. The CGH analyses of the DTC from a patient with esophageal cancer were done in context of a study that was approved by the ethics committee of the medical faculty of the Heinrich-Heine University Düsseldorf (#2316 & #2655) for which written informed consent was obtained from the patients.

### Amplification of Single Cell DNA Using MSE-PCR (WGAM)

After single cell isolation whole genomes of the cells were amplified using adapter-linker−/MSE-PCR (WGAM) as described by Klein *et al.* [10]. In brief, the single cell was transferred into 2 µl proteinase K mastermix and digested for 10 h at 42°C. Proteinase K was inactivated for 10 min at 80°C. Subsequently, the single cell DNA was restriction digested with MseI for 3 h at 37°C. Restriction enzyme was inactivated at 65°C for 5 min. Preannealed adapter nucleotides were ligated to the restriction sites of the fragmented DNA at 15°C overnight. Amplification Master Mix was added to the samples and DNA was amplified in a multistep reaction in a thermal cycler (Method 3). For increasing DNA yield a reamplification of the primary WGAM amplified DNA was performed (Method S4).

### Amplification of Single Cell DNA Using Commercial Kits (WGAN & WGAS)

Five single cells each of the cell lines, as well as from the healthy controls were amplified utilizing two different commercial kits: the Single Cell WGA Kit, WGAN (New England Biolabs) and the GenomePlex® Single Cell Whole Genome Amplification Kit, WGAS (Sigma-Aldrich) according to the manufacturer’s protocols. Briefly, single cells were isolated as described above and transferred into 4 µl Cell Extraction Buffer (WGAN) or 8 µl dH_2_O (WGAS). The detailed protocols for each amplification method are described in methods S5 and S6.

### Comparative Genomic Hybridization with Oligonucleotide Microarrays (aCGH)

aCGH analyses on oligonucleotide arrays were performed according to the manufacturer’s instructions (Agilent Oligonucleotide Array-Based CGH for Genomic DNA Analysis, Version 6.4, August 2011, G4410-90010) with slight modifications. Basically, gDNA was restriction digested to fragments of 200–500 bp, random-primed labeled with Cyanine-5/Cyanine 3-dUTP, cleaned up and hybridized on the array slides. For labeling of single cell DNA the restriction digestion step was skipped, because the DNA generated by WGA methods used in our experiments provide optimal fragment sizes for successive labeling (Figure S2).

Our standard/basic protocol was performed with each WGAM-DNA, here 2 µg DNA were labeled using random-priming. Amplified single cell DNA served as reference and both fluorescently labeled DNAs were hybridized to the 4×180k platform with a median probe spacing (MPS) of 13 kb (according to Product Note 5990-3368EN by Agilent Technologies). Additionally, experiments with the other platforms from Agilent Technologies (1×1 M, MPS = 2.1 kb; 2×400k, MPS = 5.3 kb and 8×60k, MPS = 41.4 kb) were performed. For each cell line or healthy control an aCGH experiment with the respective gDNA (1 µg) was performed according to standard conditions. The additionally tested labeling methods are described in method S7.

All CGH arrays were processed using the Microarray Scanner G2565CA by Agilent Technologies with 3 µm resolution and 16 bit color depth.

The whole aCGH optimization workflow is shown in Figure S1.

### Analysis of aCGH Data

The output image files were imported, normalized and fluorescent ratios for each probe were determined using Feature Extraction software (Agilent Technologies, Version 10.7.3.1, Protocol CGH_107_Sep09). Feature Extraction output files were imported into the Genomic Workbench 5.0.14 software. aCGH data were examined using the aberration detection method 2 (ADM-2) algorithm with a threshold of 6.0. Centralization Algorithm was set to a threshold of 4.0 with a bin size of 10. An aberration filter was defined for identifying copy number alterations, here changes only were considered as true positive events with a minimum log2ratio of ±0.25 and a minimum of 3 consecutive probes with the same polarity per region, reaching a resolution of ∼40 kb for the 4×180k arrays.

### Analyses for Quality, Sensitivity and Specificity of the aCGH Experiments

#### Specific activity (SA)

To determine the degree of labeling the value for specific activity (SA) was calculated as following: Specific Activity (pmol dyes per µg genomic DNA) = pmol per µl of dye/µg per µl genomic DNA. According to the manufacturer’s protocol (Agilent Oligonucleotide Array-Based CGH for Single Cell Analysis, June 2012, G4410-90012) values for Cy3 should reach 20–30 and for Cy5 15–25.

#### Derivative log ratio spread (DLRS)

To evaluate the performance quality in the aCGH experiments of the differently amplified single cell DNA we compared the derivative log ratio spread (DLRS), a measure of probe-to-probe noise ratio, which was generated for each array. According to the manufacturer’s manual the ideal value for gDNA ranges between 0.1–0.2 for an excellent, 0.2–0.3 for a good and >0.3 for a poor performance. For single cells the quoted value is considerably higher, ranging about <1.

#### Aberration filter

For aberration detection and reduction of background noise in aCGH experiments of the amplified samples we tested twelve aberration filters, which were used additionally to the ADM-2 algorithm. For each amplification method (WGAM, WGAN & WGAS) and each sample group, copy number regions were filtered according to the following settings: minimum of 3, 5, 10, 30, 50 or 100 consecutive probes and a minimum average absolute log2ratio of ±0.25 or ±0.5, respectively. The calls were compared to the corresponding references whose filter settings were fixed to three consecutive probes and a minimum average log2ratio of ±0.25. True positive rates (TPR) and false positive rates (FPR) were then computed for all filter parameter combinations to evaluate the filter combination most suitable for single cell aCGH copy number analyses.

#### ROC curves

To assess the sensitivity and specificity of all single cell aCGH experiments, the copy number log2ratios of every probe on each single cell array were compared to the ADM-2 copy number calls of their corresponding reference array with non-amplified gDNA of the respective cell line by means of an receiver operating characteristic (ROC) curve. The comparison refers to the presence of an alteration in the single cell data versus the reference and neither to its type (gain or loss), nor to its actual copy number. The ratios of the array probes for the single cell array experiments were smoothed by assigning the averaged ratio to each probe after a binning with ADM-2. Bins had a size of at least 3 probes and ratios were previously normalized by the ADM-2 Centralization. Replicate probes on the arrays were excluded from the analysis. The overlap with regions from the copy number calling of the reference arrays was computed to divide the probes of the single cell arrays into true and false cases. The R package ROCR [22] computed true and false positive rates for various ratio thresholds and the area under the curve (AUC).

## Results

### Establishment of an aCGH Protocol for WGAM Products from Single Cells

As a starting point to establish single cell aCGH on the 4×180k Agilent aCGH platform we combined WGAM with standard random-primed labeling utilizing the Genomic DNA Enzymatic Labeling Kit from Agilent. WGAM from single cells usually yields 5–8 µg DNA with a fragment length of 0.2 to 1 kb (Figure S2). Since this fragment length already appeared optimal for random-primed labeling, we omitted the restriction digestion step of Agilent’s standard labeling protocol, necessary in case of high molecular weight gDNA. For the labeling reaction we used 2 µg input DNA of the WGAM products from single cells, respectively.

**Figure 1:**
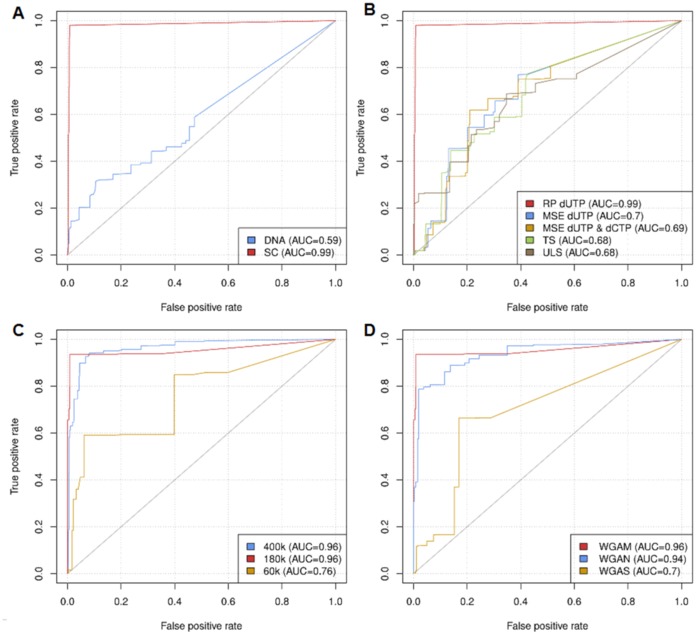
ROC curves for aCGH experiments with WGAM amplified single cells. Cell lines OE19 A) & B) and REH C) & D). An experiment performed according to standard protocol and with gDNA from each corresponding cell line served as a reference array for ROC analysis. A) Digested high molecular DNA vs. single cell amplified DNA used as reference DNA. B) Comparison of different labeling methods (RP = random-primed labeling, MSE = MSE-PCR based labeling 1 or 2, TS = Thermosequenase labeling, ULS = Universal Linkage System™). C) Comparison of aCGH platforms (2×400k, MPS = 5.3 kb, 4×180k, MPS = 13 kb and 8×60k, MPS = 41.4 kb). D) Comparison of different amplification methods (WGAM, WGAN and WGAS).

**Figure 2:**
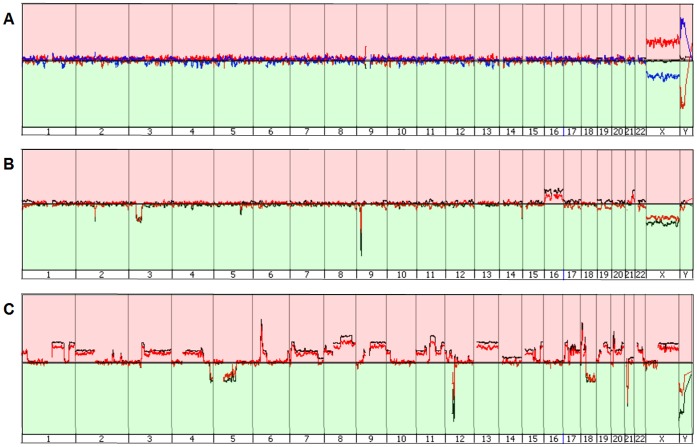
Genome wide aCGH profiles of gDNA and associated amplified single cell DNA from healthy controls, REH and OE19. Peaks upstream the baseline (red area) denote a gain, peaks downstream the baseline (green area) indicate a loss of chromosomal material. A) Healthy control black line = gDNA, red line = female single cell vs. male single cell, blue line = male vs. female single cell. B) REH black line = gDNA, red = single cell. C) OE19 black line = gDNA, red = single cell.

We compared the aCGH performance of WGAM amplified single PBMNCs to high molecular weight gDNA serving as reference DNA. This experiment clearly demonstrated that only WGAM products of single PBMNCs enabled successful aCGH. The usage of high molecular weight gDNA as a reference led to a higher rate of false positive calls (AUC: 0.59 vs. 0.99) compared to WGAM-DNA (Figure 1 A). Typical profiles retrieved from aCGH experiments using WGAM amplified single PBMNCs as reference DNA together with single cell WGAM products from different cell types as test DNA are shown in Figure 2 A–C. We noted no obvious differences in aCGH profiles from the WGAM-DNA compared to their corresponding unamplified gDNA.

Next we tested five different labeling methods for their suitability for aCGH experiments with WGAM-DNA (i.e. PCR-based methods or chemical labeling). Here, only random-rimed labeling showed satisfactory results in visual as well as in ROC analysis (Figure 1 B). The values for AUC were 0.99 for random-primed labeling compared to 0.68–0.7 for the other methods, respectively. Concerning specific activity, best results could be achieved by RP, TS and ULS labeling (Figure S3).

A decrease of input DNA in the random-primed reaction down to 1 µg did not affect the quality of aCGH (Figure S4 A). To save primary WGAM products more effectively, e.g. for additional sequence analyses, we tested the use of reamplification products for labeling and aCGH. The reamplification PCR required only 2 µl (4%) of the 50 µl primary WGAM product. No obvious differences were noted between the aCGH profiles of reamplified and primary WGAM products, respectively, as demonstrated by similar AUC values in ROC analysis (Figure S4 B).

In a next set of experiments, we checked the impact of array resolution by testing all of Agilent’s available aCGH platforms. While hybridizations of WGAM DNA (basic protocol) were successful on high-resolution platforms, with good true and false positive rates (AUC = 0.92–0.98), aCGH was impaired when the 8×60k platform was used (AUC = 0.76–0.91), shown exemplarily for the cell line REH in Figure 1 C. Visual analysis with the software Genomic Workbench also showed noisy and fuzzy profiles reflected by higher DLRS values in the analyses with the 8×60k arrays (Figure S5 A and B).

**Figure 3:**
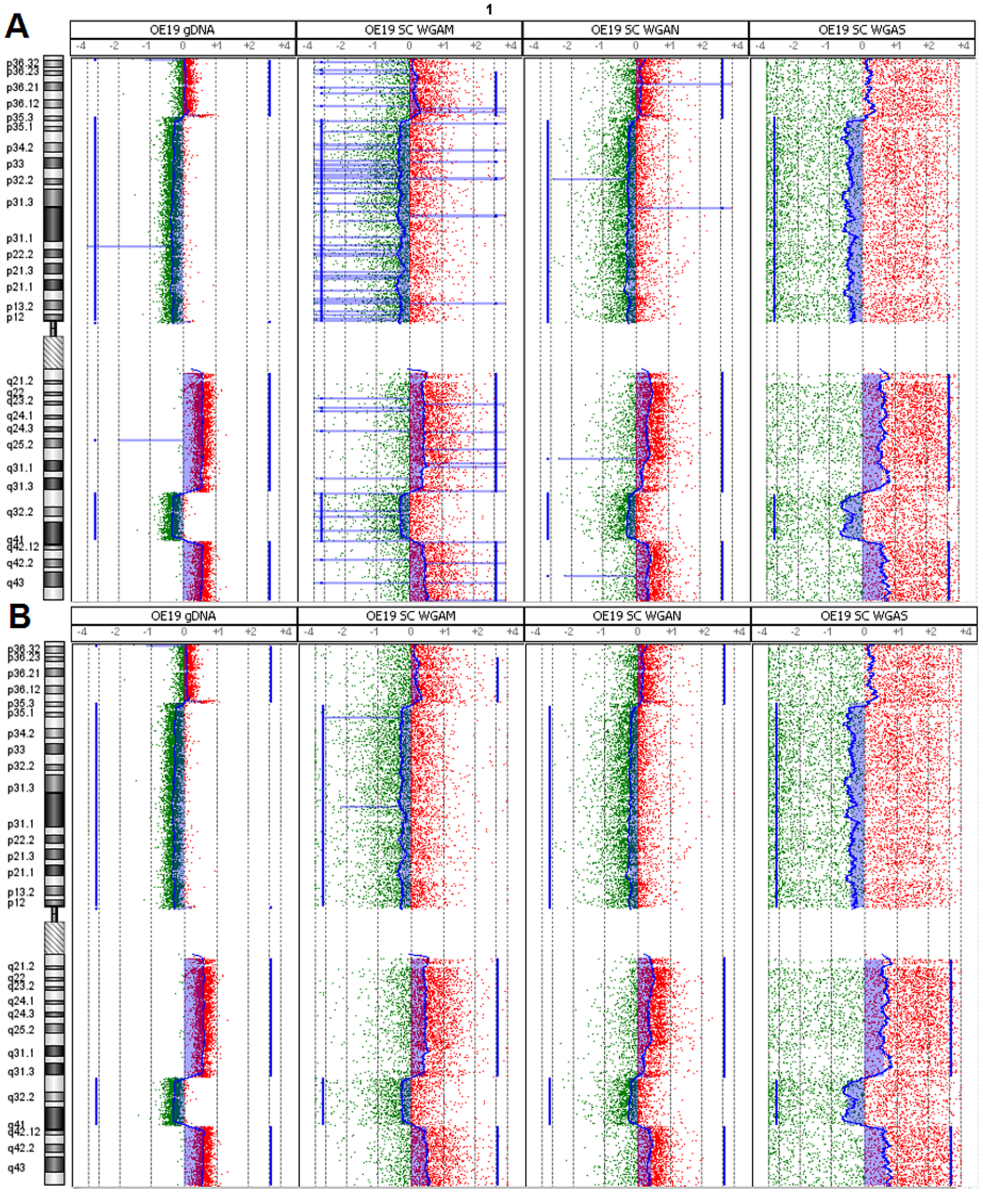
Effect of custom aberration filter. Visualization of chromosomal alterations on chromosome 1 in Genomic Workbench using aberration detection algorithm ADM-2. Blue filled areas denote a gain (right) or loss (left) of chromosomal material. A) Analysis of gDNA, WGAM-, WGAN- and WGAS-single cell amplified DNA without aberration filter. B) Analysis of the same samples with aberration filters (≥3 consecutive oligonucleotides, ≥ log2ratio ±0.25).

**Figure 4:**
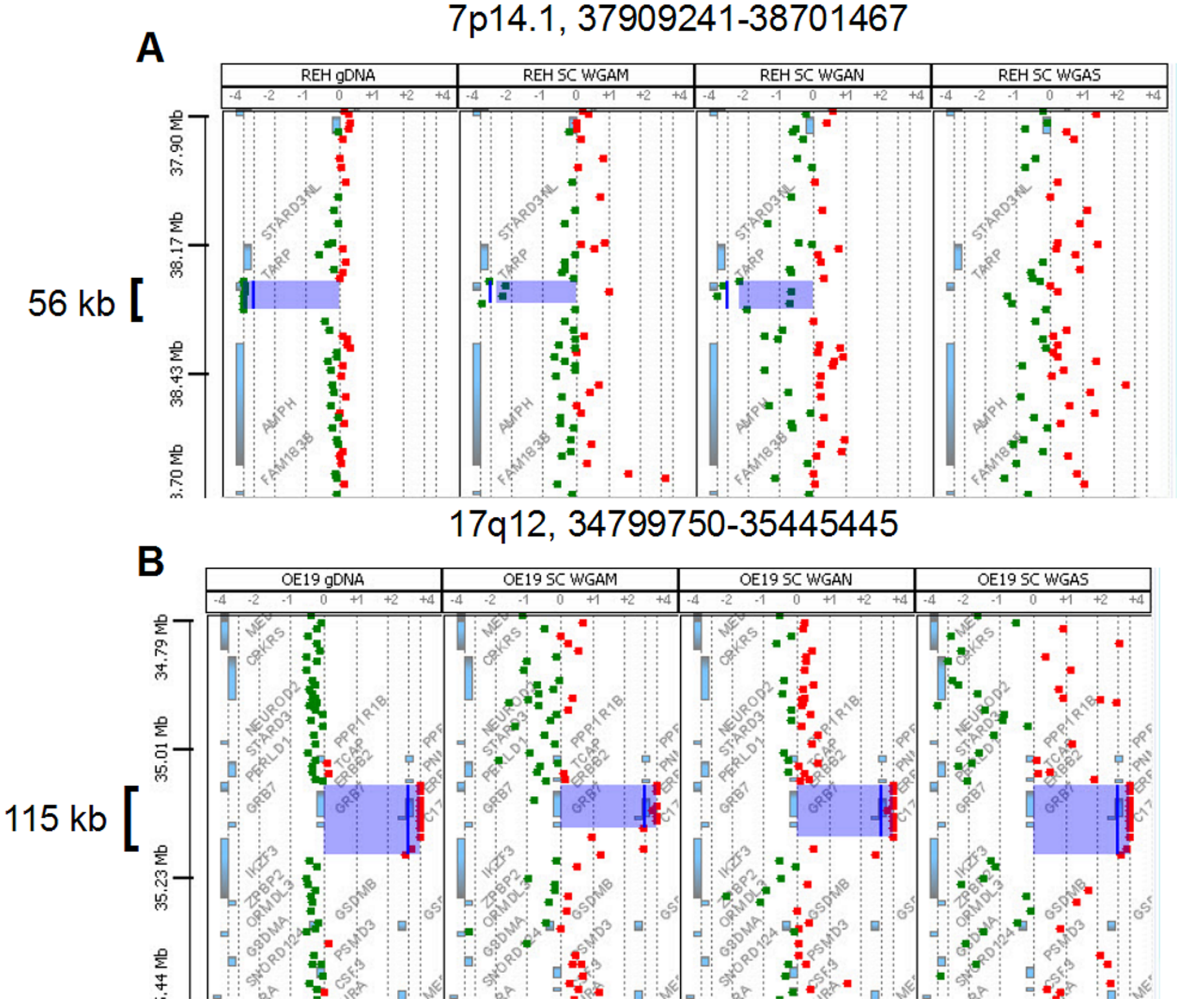
Visualization of smallest detected alterations. Smallest alteration (blue area) in gDNA and single cell amplified DNA generated with three WGA methods (WGAM, WGAN & WGAS from left to right). A) 56 kb deletion of material from chromosome 7p14.1 in cell line REH. Note that the deletion could not be retrieved in the WGAS amplified single cell. B) 115 kb gain of material from chromosome 17q12 in the cell line OE19.

For determination of a suitable custom aberration filter for copy number analysis in single cells we tested twelve different filters (Table S2 A & B). By using a custom aberration filter with three consecutive probes and ±0.25 log2ratio the FPR could already be reduced to a minimum of 0.01–0.07 (Figure 3 A & B) compared to the analysis without aberration filter (0.31–0.58). We observed no apparent difference in TPR and FPR rates using a filter with a range between 3 and 100 consecutive oligonucleotide probes (Table S2). Using this filter range (log2ratio ±0.25, minimum of 3–100 probes per region) a resolution of 40 kb–1.5 Mb could be achieved on 4×180k microarrays. Nevertheless increasing the log2ratio threshold from 0.25 to 0.5 dramatically decreased the TPR (0.980.5). By using the optimal filter settings with ±0.25 in a minimum of three consecutive probes a 56 kb deletion of the TARP gene on chromosome 7p14 in the REH WGAM-DNA (Figure 4 A) and a 115 kb amplification of ERBB2/HER2 gene on chromosome 17q12 in the OE19 WGAM-DNA (Figure 4 B) could be retrieved as the smallest chromosomal loss or gain in the tested samples.

After having established the final WGAM single cell aCGH protocol (Figure S1, green box) we determined its robustness and reliability by performing technical and biological replicates. Repeated hybridization of the same single cell at different time points revealed no relevant alterations in copy number change detection (Figure S6 A). Experiments with three different single cells from individual cell culture passages basically gave the same results and only a slight deviation in the aCGH profile with different heights of the log2ratio were noted (Figure S6 B).

**Figure 5:**
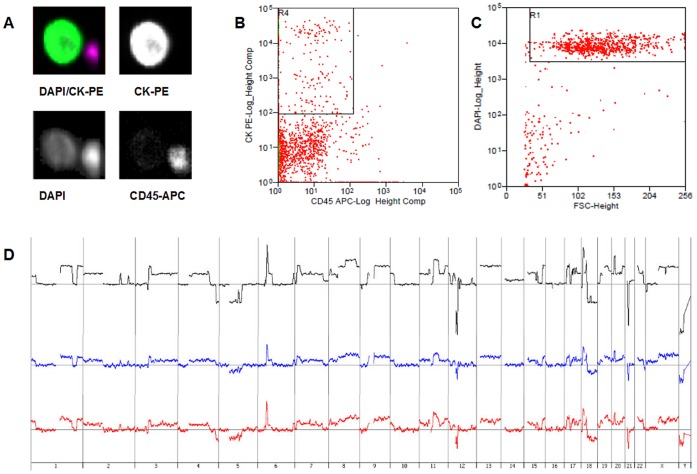
aCGH experiments with CellSearch® identified and MoFlo™ XDP sorted WGAM single cells from cell line OE19. A) Screenshot from CellSearch® System from an EpCAM captured CK^+^/DAPI^+^/CD45^−^ tumor cell: overlay of DAPI^+^/CK-PE^+^ (upper left), CK-PE^+^ (upper right), DAPI^+^ (lower left)/CD45-APC^−^ (lower right). B) & C) Dotplot from MoFlo™ XDP, CK^+^ and CD45^+^ cells (B) and DAPI^+^ cells (C). D) Overview of the genomewide profiles from two cells identified with CellSearch®, isolated with MoFlo™ XDP and amplified by WGAM. Black =  gDNA, blue = WGAM single cell #1 and red = WGAM single cell #2.

In order to test the final protocol in clinical applications we used two different settings. First, we analyzed a DTC, isolated from the bone marrow of an esophageal cancer patient, which was amplified by WGAM and investigated by conventional mCGH. We then performed aCGH from the same WGAM product according to our final protocol and observed a good concordance between the two profiles (Figure S7 A and B). However, aCGH revealed smaller alterations, which could not be identified in CGH analyses due to the low resolution of mCGH (Figure S7 C and D). In the second experiment we tested the feasibility of our protocol for another clinical application: the CellSearch® System that is commonly used for CTC identification (Method S8). To model a typical CellSearch® test we spiked the EpCAM positive cell line OE19 in 7.5 ml peripheral blood and retained the sample in a CellSave tube for 72 h at room temperature. Tumor cells were then selected, identified and enumerated using the CellSearch® System (Figure 5 A). Isolation of single cells was then performed by fluorescence activated cell sorting (FACS) utilizing a MoFlo™ XDP cell sorter (Figure 5 B and C). aCGH analysis of sorted single cells was performed as described in our final protocol (Figure S1, green box). Comparison of the aCGH profiles from two different single cells to the profile of the gDNA of the cell line showed no alterations in copy number change detection (Figure 5 D).

### Comparison of Amplification Methods

Finally, we compared our protocol (WGAM) with commercially available PCR-based whole genome amplification methods. Prior to array analyses we compared concentrations and yields after WGA, as well as the genomic representation by a multiplex-PCR covering six different genomic loci. The mean concentration of the WGA-DNA varied between 202 to 318 ng/µl for the different amplification methods (Figure S8 A). The yield of DNA ranged from 1 to 20 µg in amplified single cells (n = 60) with the highest yields observed in WGAS (11–14 µg) (Figure S8 B). Upon agarose gel electrophoresis, WGAS products showed the largest fragment sizes (0.2≥3 kb) compared to WGAM and WGAN (0.2–1 kb) (Figure S2). Thus it was not surprising that the multiplex-PCR analyses (Method S9, Table S1) to screen the primary amplification products for successful amplification revealed different allelic distribution patterns of the three WGA methods. While the amplification of the loci in nearly all of the multiplex-PCRs was highly reproducible in WGAM and WGAN (Figure S9 A & B), WGAS exhibited an irregular pattern, ranging from two to four (mean = 3) amplified loci (Figure S9 C). In our hands a successful amplification of numerous loci (>3) was linked to a good performance in aCGH analyses, especially if the larger MseI-restriction fragments (Table S1) were amplified in WGAM (data not shown). When we compared the aCGH performance of the different WGA products, we observed the best DLRS mean DLRS values (triplicates) in WGAN (0.41), followed by WGAM (0.56). The mean DLRS value for WGAS was notably higher (1.15) and in contrast to WGAM and WGAN, differed significantly (p = 0.0156) from gDNA (Figure S10). DLRS retrieved for the amplified single cell DNAs with the respective methods were above the threshold indicating a poor performance of gDNA as mentioned in the manufacturer’s manual. However, all observed DLRS values were in line with data provided in the recently published single cell protocol from Agilent (<1). Surprisingly, the WGAS method recommended by Agilent gave only poor aCGH results also reflected by a high false positive rate (AUC = 0.7) in aCGH analyses with single cell DNA from the REH cells (Figure 1 D). In contrast, the two other amplification methods WGAM and WGAN showed a comparable good result in aCGH analyses.

## Discussion

Here we present a protocol for single cell aCGH, which can detect alterations as small as 56 kb. The core methodology of our protocol is WGAM, an adapter-linker whole genome PCR published by Klein *et al.* [10]. This method proved to be a robust method for single cell genomic profiling when used together with mCGH [15], [23], [24] – currently the primary downstream analysis of WGAM products. A successful attempt analyzing WGAM products genome wide at higher resolution (∼1 Mb) has been made using an array constructed with PFGE-purified BAC probes [20]. PFGE purification was necessary to eliminate contaminating bacterial DNA that was identified to interfere with successful hybridization of WGAM products to BAC-probes. Despite this success, the laborious production process of the arrays clearly impeded a more widespread use of this interesting technology. Furthermore, the resolution of BAC-arrays is significantly inferior to that of current oligonucleotide arrays [17]. Initially published experiments combining WGAM products on 1×244k arrays (Agilent) gave poor results [20]. However, after revisiting this WGAM aCGH approach, we found two major obstacles within the original protocol used by Fuhrmann *et al.* that inhibited successful oligonucleotide aCGH. The necessary changes appear quite simple retrospectively, but were the key to use WGAM products on 4×180k oligonucleotide arrays enabling a ∼20-fold higher resolution compared to those of the PGFE-BAC arrays.

The first critical point we changed was the use of a suitable reference DNA. According to the original description, Fuhrmann *et al.* cohybridized single cell WGAM products as test-DNA together with gDNAs from 1000 non-cancer cells as reference DNAs [20]. In addition, the two DNAs were labeled with different methods (thermosequenase labeling vs. random-primed labeling). However, several reports indicated that aCGH analyses with WGA products require similarly amplified and processed reference DNA [25], [26]. This might be related to a biased amplification, which can be a general problem in PCR-based WGAs, especially due to unequally amplified GC-rich fragments [27]–[29] in adapter-mediated PCRs (WGAM & WGAN). However, in our aCGH experiments we could not observe bias-related artifacts within GC-rich regions (e.g. in the regions of bright bands of Giemsa-staining) in any of the amplification techniques, when using an equally amplified single cell reference DNA. In line with this hypothesis are the data by Arneson *et al.* [30] who reported about aCGH on 19k cDNA-Arrays with random-primed labeled WGAM products from laser-microdissected FFPE tissues. Importantly, they used samples from healthy laser-microdissected FFPE tissues as reference DNA, which were WGAM amplified and labeled in the same way as the test-DNA. In accordance, we also found that only cohybridization of single cell WGAM products, i.e. a reference-DNA similar treated as the test-DNA, enabled successful aCGH. In order to definitively clarify the fidelity of the WGA methods in terms of amplification biases resequencing of the WGA products using next generation sequencing approaches are needed.

The second important change to the original protocol concerns the labeling of the WGAM product. In our hands, the standard random-primed labeling according to the Agilent protocol with 2 µg input DNA gave the best results, producing fewer false positive and negative results in aCGH. All other tested labeling methods, including those specifically designed to label WGAM products via the adapter sequences, were less reliable and introduced artificial amplifications and deletions in the aCGH profiles resulting in lower AUC values in ROC analyses. Tsubosa *et al.* also showed, that random- primed labeling is a superior method to label DNA for aCGH experiments [31]. A major disadvantage of this method is the high amount of DNA needed for the reaction, compared to the quantity needed for the PCR-based methods, e.g. MSE-PCR labeling. Therefore, it was an important finding that labeling and hybridization of WGAM reamplification products as well as a reduced amount of 1 µg had no influence on aCGH performance. This allows efficient use of precious single cell libraries and opens up the possibility for using the WGA-DNA for multiple consecutive analyses, e.g. mutation analysis, qPCR or next-generation sequencing approaches.

Another relevant factor for successful aCGH was in fact the oligonucleotide density on the array slides. While a median probe spacing of 41.4 kb was insufficient, a median probe spacing of 13 kb or lower enabled aCGH of single cell WGAM products. In a recent publication Bi *et *al. also discovered that resolution capability of single cell analysis is directly linked to probe density [32]. However, we could not gain more information about the CNAs with the expensive high-density (up to 2 kb) arrays. So we concluded that the 4×180k platform is suitable for high-resolution analysis and is also reasonable from an economic point of view, offering the possibility to hybridize four samples simultaneously.

In order to establish a standardized workflow we used Agilent’s Genomic Workbench for data analysis. The ADM-2 algorithm, providing reliable results in CNA detection of cancer cells [33], was also appropriate to detect CNAs in the single cell experiments. Our final custom aberration filter could reduce false positive results to a minimum. The experiments with various filter settings pointed out that the TPR started to diminish noticeably at a log2ratio threshold of 0.5. Thus we recommend using an aberration filter with a threshold set to 0.25. FPR can be reduced to a minimum by setting the filter to three consecutive oligonucleotides per region with a log2ratio of 0.25, whereby a theoretical resolution of 40 kb can be achieved in the single cell aCGH experiments. In one of our tested cell lines we could retrieve a small deletion of only 56 kb using these filter settings. To our knowledge, such a high resolution has never been reported in single cell array experiments (Table S3).

Finally, we could also demonstrate that our aCGH protocol was applicable to single fixed and unfixed cells that were identified and isolated under experimental conditions found in different clinical settings. We therefore think that aCGH with WGAM products might be useful in diverse clinical applications. It is however important to note, that under other suboptimal conditions and poorer DNA quality aCGH performance and resolution might be lower.

In the biological and technical replication experiments no significant differences between the single cells could be observed depicting the high robustness and fidelity of the chosen method.

In our comparison experiments with other commercial available non-linear WGA methods (Table 1), the observed precision and robustness of WGAM could not be observed for WGAS, which is the suggested method by Agilent to perform single cell aCGH (Agilent, G4410-90012). In our hands, WGAS only gave poor results, which could explain the relatively low resolution described in the few available studies that was achieved even with high-density oligonucleotide arrays [32], [34], [35]. In the REH cell line disparity between the performances of amplification methods became apparent due to the small and distinct alterations in this cell line [36]. In contrast, WGAN seemed to be as precise and robust as WGAM. However, in contrast to the proprietary WGAN method, WGAM has the advantage of an “open-source” method for transparent designing, testing and controlling downstream analysis. In these comparison experiments the DLRS was a suitable indicator for aCGH performance. We could show that the amplification method with the worst performance, namely WGAS, also had the highest DLRS, which was significantly different to the value for gDNA, whereas no significant difference was observed for WGAM and WGAN. Notably, we observed that the DLRS values of all single cell experiments were comparable to the value (<1) given in the Agilent single cell manual (G4410-90012), in comparison to matched gDNA, all of these samples performed well in aCGH analyses and also small copy number changes could be discovered. A similar circumstance was observed for FFPE samples where aCGH performance was good despite high DLRS, e.g. a DLRS of ∼0.3 as reported by Hostetter *et al.* [37]. This indicates a general higher value in DLRS for difficult samples.

**Table 1:** Overview and comparison of non-linear PCR based whole genome amplification methods.

	WGAM	WGAN	WGAS
**Technique**	non-linear thermal cycling	non-linear thermal cycling	non-linear thermal cycling
**Input DNA** *	SC/6.5 pg	SC/6.5 pg	SC/6.5 pg
**Output**	5–8 µg^†^	5–8 µg^†^	11–14 µg^†^
**Concentration**	∼240 ng/µl^†^	∼240 ng/µl^†^	∼260 ng/µl^†^
**Product length**	0.2–1kb^†^	0.2–1kb^†^	0.2≥3kb^†^
**Time required**	3 days	2.7 h	5 h
**Handling**	elaborate	moderate	moderate
**Costs**	low	high	moderate
**multiplex-PCR**	5/6	5/6	2–4/6
**aCGH applicability**	yes	yes	no

* according to manufacturer’s instructions, ^†^as observed in this study, SC = single cell.

In conclusion, we could show that single cell WGAM combined with random-primed labeling enables aCGH at a relatively high resolution comparable to those reported currently for single cell next-generation sequencing (NGS) approaches [38], [39], [40]. The provided aCGH protocol enables a relatively fast and cost-effective way to characterize CNAs on the single cell level. Furthermore, since only a minute amount of the WGAM product is needed for aCGH, further comprehensive analyses e.g. sequence or mutational analysis can be performed subsequently, allowing a deeper insight into single cell genomes.

## Supporting Information

Figure S1 **Workflow of aCGH optimization process.** Variegated parameters of the basic protocol are shown in the upper box. The green box shows the final protocol. The optimized protocol was used for comparison of the three amplification methods, namely WGAM, WGAN and WGAS.
(TIF)

Figure S2 **Agarose gel analysis (0.8%) of WGA single cell DNA.** 1 =  WGAM, 2 =  WGAM reamplified, 3 =  WGAN, 4 =  WGAS, M = marker. The fragment sizes of the WGAM and WGAN amplified single cells ranges between 0.2–1 kb, whereas the fragment size of the WGAS amplified cells varies between 0.2≥3 kb. Reamplification of the WGAM product slightly reduces fragment size to 0.1–0.5 kb.
(TIF)

Figure S3 **Comparison of value for specific activity for the different labeling techniques.** Specific activity (SA = pmol dyes/µg DNA) of five differently labeled WGAM-DNAs of a representative experiment from cell line OE19 in comparison to the corresponding gDNA labeled according to the manufacturer’s protocol. green bars = SA Cyanine-3-dUTP, red bars = SA Cyanine-5-dUTP.
(TIF)

Figure S4 **ROC curves for aCGH experiments with WGAM amplified single cells.** a) amount of input DNA in aCGH experiment 1 vs. 2 µg (healthy control). b) primary WGAM product vs. reamplified WGAM product (OE19). An experiment with the corresponding gDNA was performed according to the manufacturer’s protocol and served as a reference array for ROC analysis.
(TIF)

Figure S5 **aCGH experiments with WGAM-DNA hybridized to different platforms.** a) Comparison of performance of WGAM single cell DNA from the cell line OE19 hybridized to different aCGH platforms displayed in ascending resolutions. Chromosomal alterations on chromosome 1 were visualized with the software Genomic Workbench using aberration algorithm ADM-2. b) DLRS values of aCGH experiments with OE19 WGAM single cell DNA hybridized to different platforms compared to the reference array with gDNA from the corresponding cell line treated according to the manufacturer’s protocol. If applicable mean and standard deviation was calculated.
(TIF)

Figure S6 **Overview of genome wide aCGH profiles.** a) technical replicates, b) biological replicates of WGAM single cells from OE19. Black = gDNA, blue = WGAM single cell #1, red = WGAM single cell #2, green = WGAM single cell #3.
(TIF)

Figure S7 **Comparison of CGH and aCGH profile from a WGAM-DTC from a patient with esophageal cancer.** a) CGH profile, b) aCGH profile, c) magnification of chromosome 1 of CGH (right) and aCGH profile (left) and d) magnification of chromosome 5 of CGH (right) and aCGH profile (left). Green bars = chromosomal gain, red bars =  chromosomal loss. Differences in aCGH profile and CGH profile result from higher resolution of the aCGH platform.
(TIF)

Figure S8 **Quantitative measurements of WGA-DNA.** a) mean concentration (ng/µl) and b) mean yield (µg) of five WGAM, WGAN and WGAS amplified single cells per female and male healthy control, OE19 and REH, respectively.
(TIF)

Figure S9 **Agarose gel analysis (1.5%) of the multiplex-PCR products from differently amplified single cells.** a) WGAM, b) WGAN, c) WGAS amplified single cells from OE19 (1–3), REH (4–6) and healthy controls (7–9). 10 =  positive control, 11 =  negative control and M = marker. Please note that CADPS contains a MseI restriction digestion site, which usually prohibits successful amplification of this locus in WGAM products.
(TIF)

Figure S10 **Comparison of DLRS values of aCGH experiments with the miscellaneous WGA techniques.** Mean DLRS of aCGH experiments with three WGA single cell DNAs each, amplified by the different techniques (WGAM, WGAN and WGAS) and labeled and hybridized according to our revised protocol compared to the reference array with corresponding gDNA treated according to the manufacturer’s protocol. p-value was determined using Kruskal-Wallis and Dunn’s multiple comparsion test.
(TIF)

Table S1 Chromosomal location, sequence (according to Knijnenburg *et al.)*, primer concentration and product size of the primer pairs used in the multiplex-PCR.
(PDF)

Table S2 Evaluation experiment for a suitable aberration filter for aCGH copy number analyses in single cells.
(PDF)

Table S3 Comparison of theoretical resolution and size of detected alterations in whole genome amplified single cell aCGH experiments.
(PDF)

Method S1 Cell culture.
(PDF)

Method S2 DNA Preparation.
(PDF)

Method S3 Whole genome amplification of single cell DNA using MSE-PCR.
(PDF)

Method S4 Reamplification of primary MSE-PCR products.
(PDF)

Method S5 Whole genome amplification of single cell DNA using the Single Cell WGA Kit (WGAN, New England Biolabs, E2620S/L).
(PDF)

Method S6 Whole genome amplification of single cell DNA using Single Cell WGA Kit (WGAS, Sigma-Aldrich®, WGA4).
(PDF)

Method S7 Comparison of labeling techniques in aCGH analyses.
(PDF)

Method S8 CellSearch® System identification, MoFlo™ XDP isolation and WGAM amplification of single tumor cells.
(PDF)

Method S9 Qualitative and quantitative control of whole genome amplified DNA.
(PDF)
