# Supplementary figures and images for: A Robust Method to Analyze Copy Number Alterations of Less than 100 kb in Single Cells Using Oligonucleotide Array CGH
Source: PLoS One. 2013 Jun 25;8(6):e67031. doi: 10.1371/journal.pone.0067031 (PMC3692546; doi:10.1371/journal.pone.0067031)

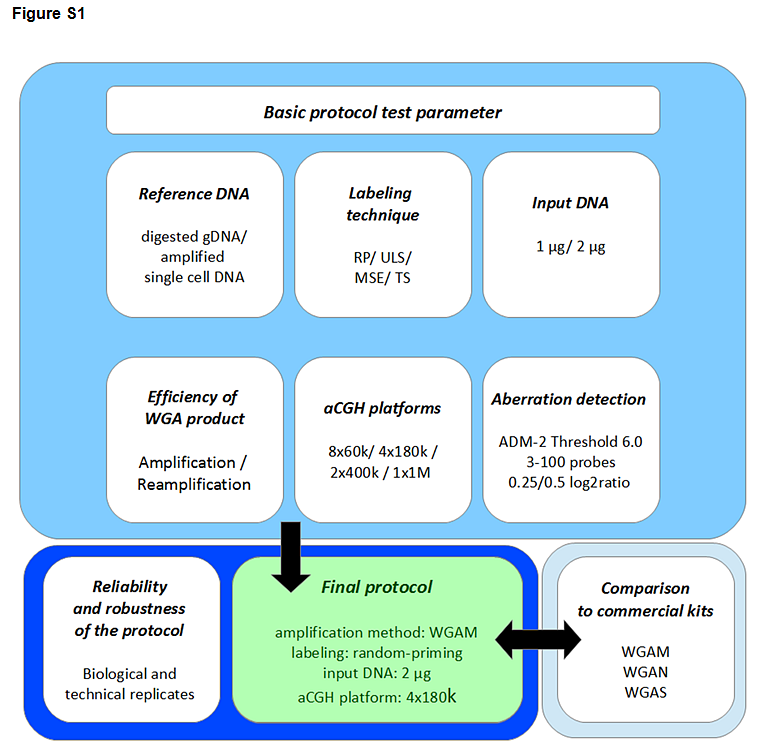

Supplement: Figure S1 — Workflow of aCGH optimization process. Variegated parameters of the basic protocol are shown in the upper box. The green box shows the final protocol. The optimized protocol was used for comparison of the three amplification methods, namely WGAM, WGAN and WGAS. (TIF) [file pone.0067031.s001.tif]

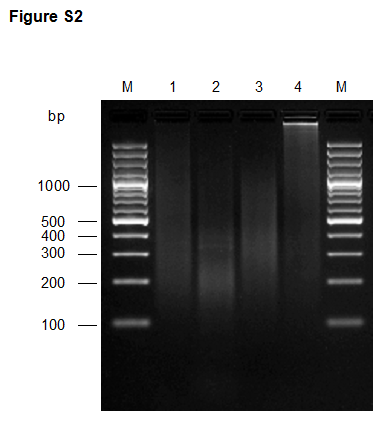

Supplement: Figure S2 — Agarose gel analysis (0.8%) of WGA single cell DNA. 1 = WGAM, 2 = WGAM reamplified, 3 = WGAN, 4 = WGAS, M = marker. The fragment sizes of the WGAM and WGAN amplified single cells ranges between 0.2–1 kb, whereas the fragment size of the WGAS amplified cells varies between 0.2≥3 kb. Reamplification of the WGAM product slightly reduces fragment size to 0.1–0.5 kb. (TIF) [file pone.0067031.s002.tif]

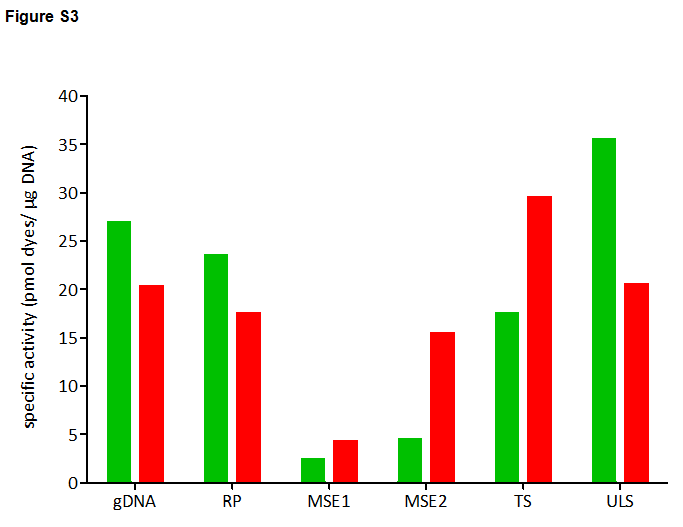

Supplement: Figure S3 — Comparison of value for specific activity for the different labeling techniques. Specific activity (SA = pmol dyes/µg DNA) of five differently labeled WGAM-DNAs of a representative experiment from cell line OE19 in comparison to the corresponding gDNA labeled according to the manufacturer’s protocol. green bars = SA Cyanine-3-dUTP, red bars = SA Cyanine-5-dUTP. (TIF) [file pone.0067031.s003.tif]

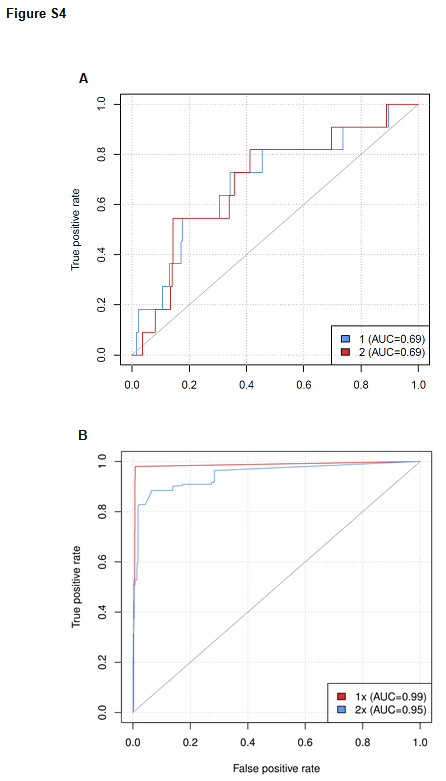

Supplement: Figure S4 — ROC curves for aCGH experiments with WGAM amplified single cells. a) amount of input DNA in aCGH experiment 1 vs. 2 µg (healthy control). b) primary WGAM product vs. reamplified WGAM product (OE19). An experiment with the corresponding gDNA was performed according to the manufacturer’s protocol and served as a reference array for ROC analysis. (TIF) [file pone.0067031.s004.tif]

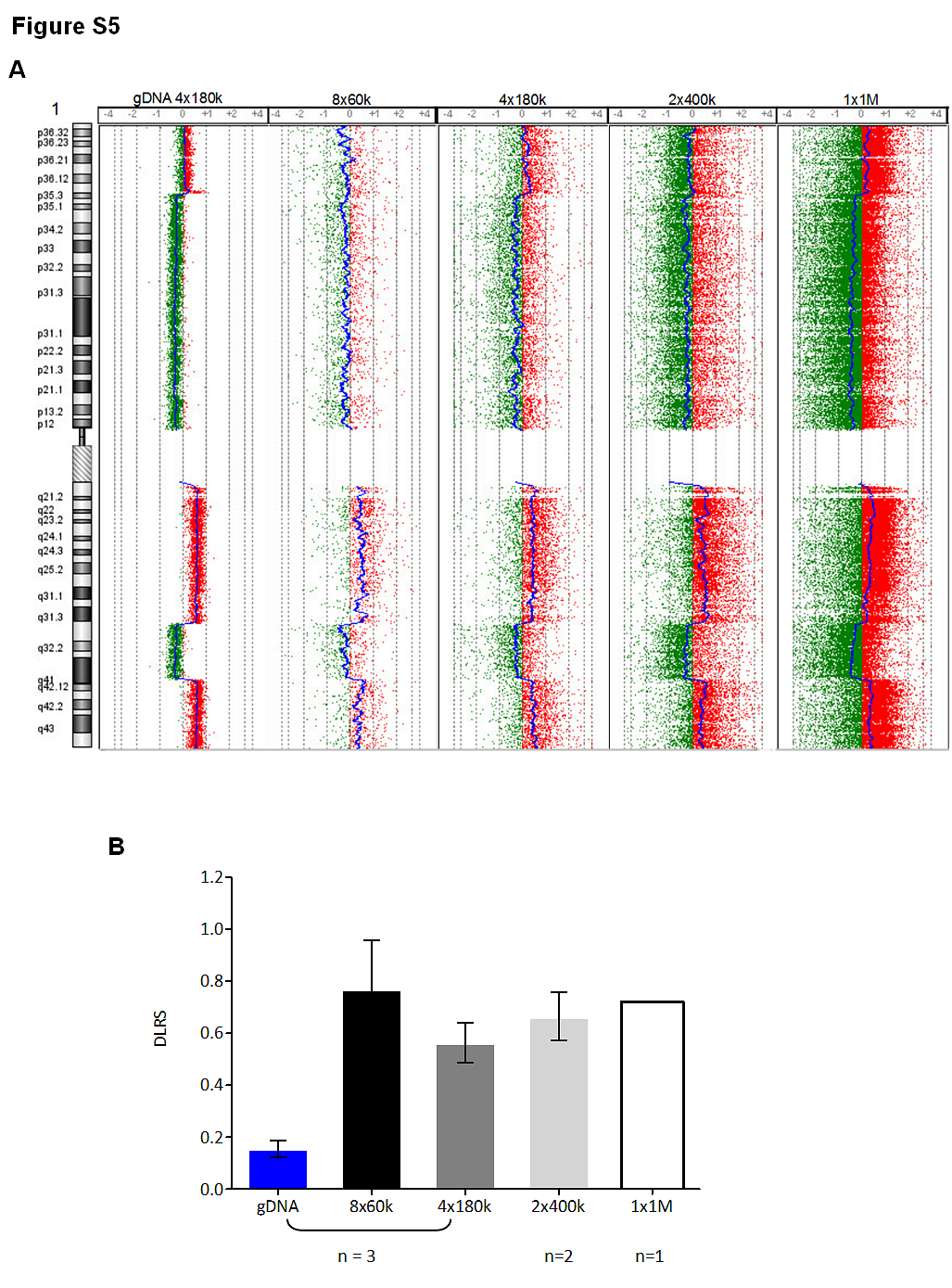

Supplement: Figure S5 — aCGH experiments with WGAM-DNA hybridized to different platforms. a) Comparison of performance of WGAM single cell DNA from the cell line OE19 hybridized to different aCGH platforms displayed in ascending resolutions. Chromosomal alterations on chromosome 1 were visualized with the software Genomic Workbench using aberration algorithm ADM-2. b) DLRS values of aCGH experiments with OE19 WGAM single cell DNA hybridized to different platforms compared to the reference array with gDNA from the corresponding cell line treated according to the manufacturer’s protocol. If applicable mean and standard deviation was calculated. (TIF) [file pone.0067031.s005.tif]

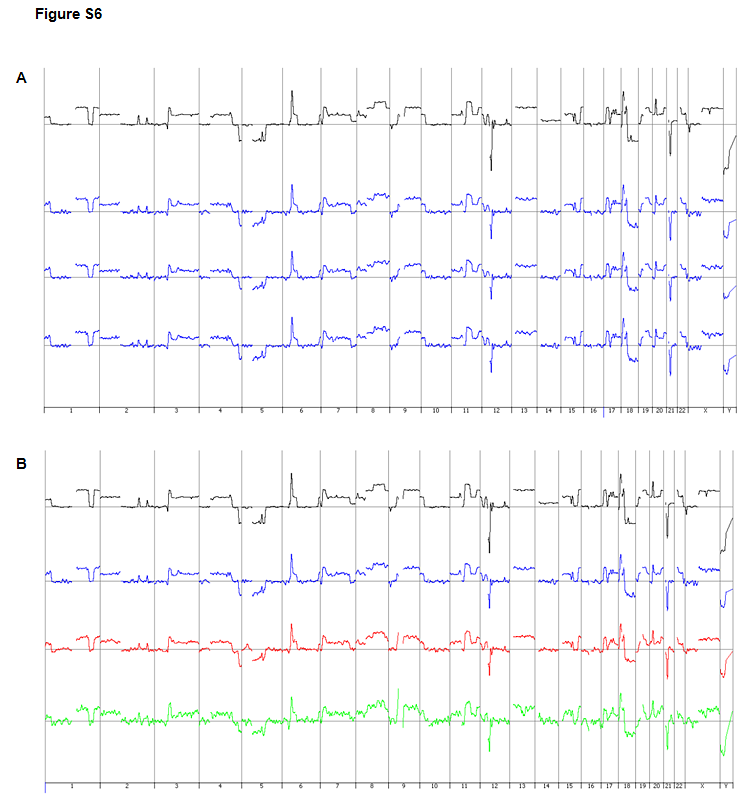

Supplement: Figure S6 — Overview of genome wide aCGH profiles. a) technical replicates, b) biological replicates of WGAM single cells from OE19. Black = gDNA, blue = WGAM single cell #1, red = WGAM single cell #2, green = WGAM single cell #3. (TIF) [file pone.0067031.s006.tif]

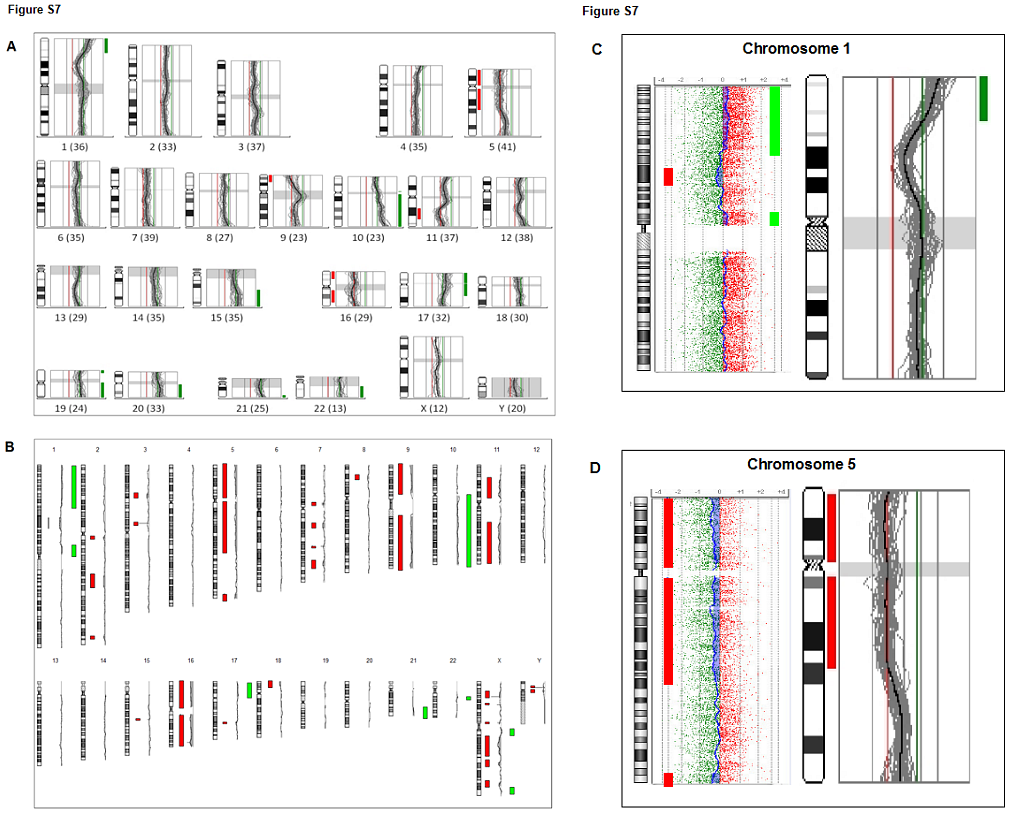

Supplement: Figure S7 — Comparison of CGH and aCGH profile from a WGAM-DTC from a patient with esophageal cancer. a) CGH profile, b) aCGH profile, c) magnification of chromosome 1 of CGH (right) and aCGH profile (left) and d) magnification of chromosome 5 of CGH (right) and aCGH profile (left). Green bars = chromosomal gain, red bars = chromosomal loss. Differences in aCGH profile and CGH profile result from higher resolution of the aCGH platform. (TIF) [file pone.0067031.s007.tif]

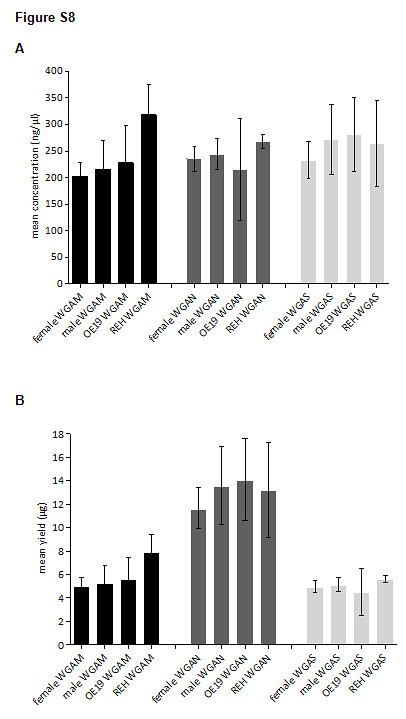

Supplement: Figure S8 — Quantitative measurements of WGA-DNA. a) mean concentration (ng/µl) and b) mean yield (µg) of five WGAM, WGAN and WGAS amplified single cells per female and male healthy control, OE19 and REH, respectively. (TIF) [file pone.0067031.s008.tif]

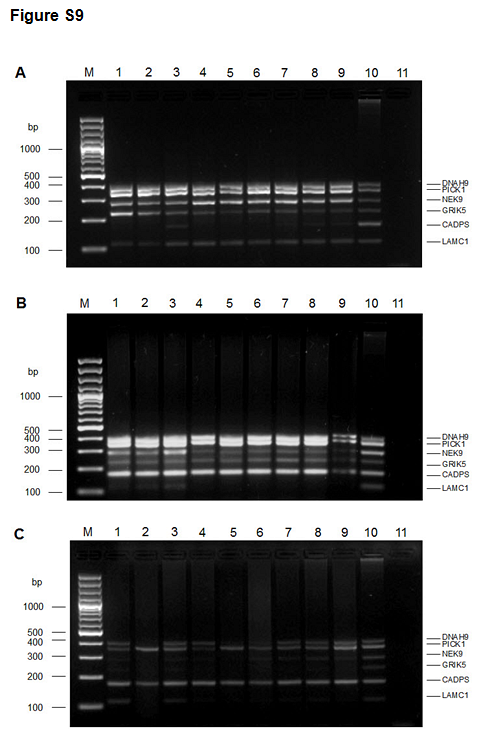

Supplement: Figure S9 — Agarose gel analysis (1.5%) of the multiplex-PCR products from differently amplified single cells. a) WGAM, b) WGAN, c) WGAS amplified single cells from OE19 (1–3), REH (4–6) and healthy controls (7–9). 10 = positive control, 11 = negative control and M = marker. Please note that CADPS contains a MseI restriction digestion site, which usually prohibits successful amplification of this locus in WGAM products. (TIF) [file pone.0067031.s009.tif]

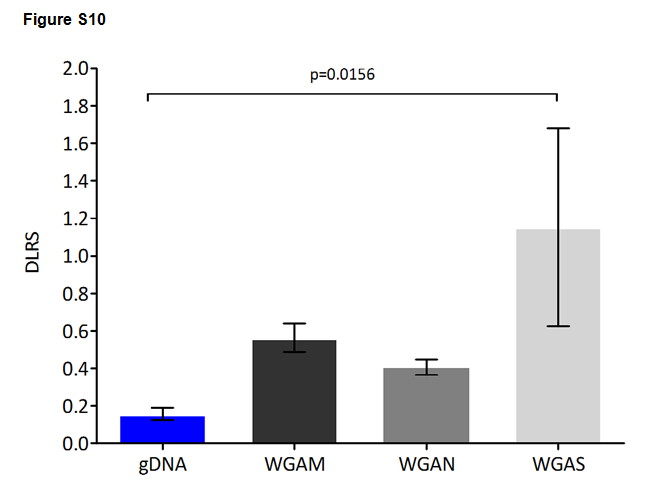

Supplement: Figure S10 — Comparison of DLRS values of aCGH experiments with the miscellaneous WGA techniques. Mean DLRS of aCGH experiments with three WGA single cell DNAs each, amplified by the different techniques (WGAM, WGAN and WGAS) and labeled and hybridized according to our revised protocol compared to the reference array with corresponding gDNA treated according to the manufacturer’s protocol. p-value was determined using Kruskal-Wallis and Dunn’s multiple comparsion test. (TIF) [file pone.0067031.s010.tif]
