# Supplementary material for: A Robust Method to Analyze Copy Number Alterations of Less than 100 kb in Single Cells Using Oligonucleotide Array CGH
Source: PLoS One. 2013 Jun 25;8(6):e67031. doi: 10.1371/journal.pone.0067031 (PMC3692546; doi:10.1371/journal.pone.0067031)
Supplement: Table S1 — Chromosomal location, sequence (according to Knijnenburg et al.), primer concentration and product size of the primer pairs used in the multiplex-PCR. (PDF) [file pone.0067031.s011.pdf]

Table S1. Chromosomal location, sequence (according to Knijnenburg *et al.)*, primer concentration and product size of the primer pairs used in the multiplex-PCR

Gene	Chromosomal location	Sequence	Concentration†	Product Size	AT/GC content(%)	WGAM fragment size
LAMC1	1:182.992.595-183.114.727	FWD: tctgctttgggcattcttct<br>REV: ttctaacaggttgggggatg	0.2 µM	111 bp	57.6/42.3	171 bp
CADPS*	3:62.384.021-62.861.064	FWD: ccccacccttcttcactaca<br>REV: gtgtgcacataccaccgaag	0.08 µM	175 bp	51.4/40.5	717 bp
GRIK5	19:42.502.73-42.569.9957	FWD: ctagctcccaccaacctcag<br>REV: ctcgatgatcccgttgatct	0.1 µM	232 bp	39.2/60.7	2520 bp
NEK9	14:75.548.818-75.593.778	FWD: gcaggagggaacctgtatga<br>REV: caggaaagaaagcccacaga	0.1 µM	288 bp	60.4/39.5	490 bp
PICK1	22:38.453.262-38.471.708	FWD: tcgtatgctggagtcctgtg<br>REV: gggatggctttgttgaggta	0.08 µM	358 bp	41.3/58.6	1111 bp
DNAH9	17:11.501.748-11.873.065	FWD: gggtctcatcaccagcattt<br>REV: gccatcttccacatggtctt	0.08 µM	401 bp	45.1/54.8	625 bp

*MseI restriction digestion site (5`T^TAA3`) in PCR product might prohibit successful amplification of this locus in WGAM products.
†final concentration of forward and reverse primer in multiplex-PCR reaction.
