# Supplementary material for: A Robust Method to Analyze Copy Number Alterations of Less than 100 kb in Single Cells Using Oligonucleotide Array CGH
Source: PLoS One. 2013 Jun 25;8(6):e67031. doi: 10.1371/journal.pone.0067031 (PMC3692546; doi:10.1371/journal.pone.0067031)
Supplement: Table S2 — Evaluation experiment for a suitable aberration filter for aCGH copy number analyses in single cells. (PDF) [file pone.0067031.s012.pdf]

Table S2. Evaluation experiment for a suitable aberration filter for aCGH copy number analyses in single cells

A

OE19	WGAM		WGAN		WGAS	
Aberration filter	TPR	FPR	TPR	FPR	TPR	FPR
NONE	0.97	0.01	0.99	0.33	0.97	0.08
minProbes=3. minAvgAbsLogRatio=0.25*	0.97	0.01	0.96	0.03	0.97	0.03
minProbes=3. minAvgAbsLogRatio=0.5	0.53	0	0.51	0.02	0.92	0.01
minProbes=5. minAvgAbsLogRatio=0.25	0.97	0.01	0.96	0.03	0.97	0.03
minProbes=5. minAvgAbsLogRatio=0.5	0.53	0	0.51	0.02	0.92	0.01
minProbes=10. minAvgAbsLogRatio=0.25	0.97	0.01	0.96	0.03	0.97	0.03
minProbes=10. minAvgAbsLogRatio=0.5	0.53	0	0.51	0.02	0.92	0.01
minProbes=30. minAvgAbsLogRatio=0.25	0.97	0.01	0.96	0.03	0.97	0.03
minProbes=30. minAvgAbsLogRatio=0.5	0.53	0	0.51	0.02	0.92	0.01
minProbes=50. minAvgAbsLogRatio=0.25	0.97	0.01	0.96	0.03	0.97	0.03
minProbes=50. minAvgAbsLogRatio=0.5	0.53	0	0.51	0.02	0.92	0.01
minProbes=100. minAvgAbsLogRatio=0.25	0.96	0	0.95	0.03	0.96	0.03
minProbes=100. minAvgAbsLogRatio=0.5	0.52	0	0.5	0.02	0.92	0.01

B

REH	WGAM		WGAN		WGAS	
Aberration filter	TPR	FPR	TPR	FPR	TPR	FPR
NONE	0.96	0.31	0.98	0.58	0.37	0.44
minProbes=3. minAvgAbsLogRatio=0.25*	0.96	0.01	0.98	0.07	0.09	0.03
minProbes=3. minAvgAbsLogRatio=0.5	0.66	0	0.97	0	0	0
minProbes=5. minAvgAbsLogRatio=0.25	0.96	0.01	0.98	0.07	0.09	0.03
minProbes=5. minAvgAbsLogRatio=0.5	0.66	0	0.97	0	0	0
minProbes=10. minAvgAbsLogRatio=0.25	0.96	0.01	0.98	0.07	0.09	0.03
minProbes=10. minAvgAbsLogRatio=0.5	0.66	0	0.97	0	0	0
minProbes=30. minAvgAbsLogRatio=0.25	0.95	0.01	0.97	0.07	0.09	0.03
minProbes=30. minAvgAbsLogRatio=0.5	0.66	0	0.96	0	0	0
minProbes=50. minAvgAbsLogRatio=0.25	0.94	0.01	0.96	0.07	0.09	0.03
minProbes=50. minAvgAbsLogRatio=0.5	0.65	0	0.95	0	0	0
minProbes=100. minAvgAbsLogRatio=0.25	0.94	0.01	0.96	0.07	0.09	0.03
minProbes=100. minAvgAbsLogRatio=0.5	0.65	0	0.95	0	0	0

This tables show all filter parameters (minimum number of x oligonucleotides and minimum log2ratio ± 0.25/0.5) and the corresponding true positive and false positive rates (TPR and FPR). The calls were compared to the corresponding references with standard filter settings. Filtering regarding the number of oligonucleotides had no notable influence on TPR and FPR, while TPR dramatically decreased when filtering alterations in terms of log2ratio above -0.5/+0.5. The optimal filter setting is marked with an asterisk (*). minProbes = minimum number of oligonucleotides, minAvgAbsLogRatio = minimum absolute log2ratio.
