# Supplementary material for: A Robust Method to Analyze Copy Number Alterations of Less than 100 kb in Single Cells Using Oligonucleotide Array CGH
Source: PLoS One. 2013 Jun 25;8(6):e67031. doi: 10.1371/journal.pone.0067031 (PMC3692546; doi:10.1371/journal.pone.0067031)
Supplement: Table S3 — Comparison of theoretical resolution and size of detected alterations in whole genome amplified single cell aCGH experiments. (PDF) [file pone.0067031.s013.pdf]

**Table S3. Comparison of theoretical resolution and size of detected alterations in whole genome amplified single cell aCGH experiments**

| WGA method | Analysis method | Theoretical resolution | Detected Aberration+ | Reference |
|---|---|---|---|---|
| GenomiPhi DNA Amplification Kit | 4k BAC array | ~820 kb* | 34.0 Mb | Le Caignec *et al.* 2006 [43] |
| GenomePlex® Single Cell Whole Genome Amplification Kit | 32k full-tiling BAC array | tiling | 8.3 Mb | Fiegler *et al.* 2007 [34] |
| Adapter-linker-/MSE-PCR | 3k BAC array | 1 Mb§ | 4.4-5.0 Mb | Fuhrmann *et al.* 2008 [20] |
| GenomePlex® Single Cell Whole Genome Amplification Kit | 2.1M whole Genome tiling array (NimbleGen) | ~5 kb* | 2.6-3.0 Mb | Geigl *et al.* 2009 [35] |
| GenomiPhi DNA Amplification Kit | 244k oligonucleotide array (Agilent) | 9 kb* | n.s. | Cheng *et al.* 2011 [26] |
| GenomePlex® Single Cell Whole Genome Amplification Kit | 244k oligonucleotide array (Agilent) | 9 kb* | 2.0-3.0 Mb | Mathiesen *et al.* 2011 [44] |
| GenomePlex® Single Cell Whole Genome Amplification Kit | 1 Mb BAC/PAC array | 1 Mb§ | 3.3 Mb | Hjortland *et al.* 2011 [45] |
| PicoPlex single cell WGA kit | 44k oligonucleotide array (Agilent) | 43 kb* | 1.2 Mb | Bi *et al.* 2012 [32] |
| GenomiPhi DNA Amplification Kit | 3k BAC array (BluGnome) | 1 Mb§ | n.s. | Konings *et al.* 2012 [46] |
| GenomePlex® Single Cell Whole Genome Amplification Kit | NGS - massively parallel sequencing (Illumina) | 54 kb§ | n.s. | Navin *et al.* 2011 [38] |

*according to manufacturer`s information or §author`s information (resolution might be lower due to certain analytical algorithms used in the experiments), +as described in the publication, n.s. = not specified.
