# Supplementary material for: A Robust Method to Analyze Copy Number Alterations of Less than 100 kb in Single Cells Using Oligonucleotide Array CGH
Source: PLoS One. 2013 Jun 25;8(6):e67031. doi: 10.1371/journal.pone.0067031 (PMC3692546; doi:10.1371/journal.pone.0067031)
Supplement: Method S1 — Cell culture. (PDF) [file pone.0067031.s014.pdf]

Method S1. Cell culture

The B-cell precursor leukemia cell line REH (DSMZ, no. ACC-22) and the esophageal adenocarcinoma cell line OE19 (DMSZ, no. ACC-700) were cultured in RPMI 1640 + Glutamax™-I supplemented with 10% fetal calf serum (FCS, both Gibco®/Life Technologies™). Cells were maintained at 37°C and 5% CO_2.
