# Supplementary material for: A Robust Method to Analyze Copy Number Alterations of Less than 100 kb in Single Cells Using Oligonucleotide Array CGH
Source: PLoS One. 2013 Jun 25;8(6):e67031. doi: 10.1371/journal.pone.0067031 (PMC3692546; doi:10.1371/journal.pone.0067031)
Supplement: Method S2 — DNA Preparation. (PDF) [file pone.0067031.s015.pdf]

Method S2. DNA Preparation

Genomic DNA (gDNA) from REH, OE19, a female (BM) and a male (NHS) healthy control (hc) were prepared using the QIAamp DNA Blood Midi Kit (Qiagen) according to the manufacturer`s protocol. DNA quality was monitored on a 1.5% agarose gel and DNA concentration was determined using Infinite® 200 PRO NanoQuant spectrometer (Tecan Group Ltd.).
