# Supplementary material for: A Robust Method to Analyze Copy Number Alterations of Less than 100 kb in Single Cells Using Oligonucleotide Array CGH
Source: PLoS One. 2013 Jun 25;8(6):e67031. doi: 10.1371/journal.pone.0067031 (PMC3692546; doi:10.1371/journal.pone.0067031)
Supplement: Method S3 — Whole genome amplification of single cell DNA using MSE-PCR. (PDF) [file pone.0067031.s016.pdf]

Method S3. Whole genome amplification of single cell DNA using MSE-PCR according to Klein *et al.* 1999 [1] with modifications according to Stoecklein *et al.* 2002 [41]

Reagents:

- Proteinase K digestion:
  - OPA buffer:
    10 mM Tris acetate, ph 7.5(Fluka, 93337)
    10 mM Magnesium acetate (Fluka, 63052)
    50 mM Potassium acetate (Fluka, 95843)
  - Igepal CA-630 (Sigma, 18896)
  - Tween-20 (Sigma, P9416)
  - Proteinase K (Roche, 03115828001)
  - Nuklease-free water (Gibco, 10977015)
- MSE digestion:
  - OPA buffer (see above)
  - Mse I hc (NEB, R0525M)
  - Nuklease-free water (Gibco, 10977015)
- Preannealing:
  - OPA buffer (see above)
  - LIB1 (MWG 5´-AGTGGGATTCCTGCTGTCAGT-3´)
  - ddMSE11 (MWG 5´-TAACTGACAGCdd-3´)
  - Nuklease-free water (Gibco, 10977015)
- Ligation:
  - T4-DNA-Ligase hc (Roche, 10799009001)
  - ATP 40 µmol Conc. 100 mM (special quality for molecular biology) (Roche, 11140965001)
- Primary PCR:
  - Expand Long Template PCR System (DNA PolMix + Puffer 1) (Roche, 11681842001)
  - dNTPs (GE healthcare, illustra dNTP Set, 4x100µM, 28-4065-52)
  - Nuklease-free water (Gibco, 10977015)

Isolation of single cells and Proteinase K digestion:

- Mastermix:
  Rx 10x
  2.0 µl OPA buffer
  1.3 µl Tween (10%)
  1.3 µl Igepal (10%)
  2.6 µl Proteinase K (10 mg/ml)
  12.8 µl H_2O

- Coat the bottom of a petri dish with FCS, dilute cell suspensions with 1x PBS to achieve a density of one cell per visual field under an inverse microscope with 100x magnification
- Use 1 µl pipette to isolate a single cell
- Transfer single cell into 2 µl of PK digestion mastermix
- Digest for 10 h at 42°C
- Inactivate enzyme at 80°C for 10 min

MSE digestion:

- Mastermix
  Rx 10x
  2.0 µl OPA buffer
  2.0 µl MseI (high conc., 10 U)
  16.0 µl H_2O

- Add 2 µl to each reaction tube
- Digest for 3 h at 37°C
- Inactivate enzyme at 65°C for 5 min

Preannealing of the adapters: (simultaneously to MSE digestion)

- Mastermix
  Rx 10x
  5.0 µl OPA buffer
  5.0 µl LIB1 (100 µM)
  5.0 µl ddMse11 (100 µM)
  15.0 µl H_2O

- Cycler program: 65°C-15°C (Ramp of 1°C/min)

Ligation:

- To each 3.0 µl preannealed adapters add:
  1.0 µl 10 mM ATP
  1.0 µl T4-DNA-Ligase
- Add 5.0 µl of ligation master mix to each MSE-digested cell
- Ligation 10-16 h at 15°C

Primary PCR:

- Mastermix
  3.0 µl Buffer 1
  2.0 µl dNTP-Mix (10 µM)
  35.0 µl H_2O
  1.0 µl DNA PolMix

- Add 40 µl mastermix to each sample
- Thermal cycler program:

68°C	3 min	
94°C	40 sec	14 x
57°C	30 sec	
68°C	1 min 30 sec + 1 sec/cycle	
94°C	40 sec	8 x
57°C	30 sec + 1°C/cycle	
68°C	1 min 45 sec + 1 sec/cycle	
94°C	40 sec	22 x
65°C	30 sec	
68°C	1 min 53 sec + 1 sec/cycle	
68°C	3 min 40 sec	

Store PCR products at -20°C
