# Supplementary material for: A Robust Method to Analyze Copy Number Alterations of Less than 100 kb in Single Cells Using Oligonucleotide Array CGH
Source: PLoS One. 2013 Jun 25;8(6):e67031. doi: 10.1371/journal.pone.0067031 (PMC3692546; doi:10.1371/journal.pone.0067031)
Supplement: Method S4 — Reamplification of primary MSE-PCR products. (PDF) [file pone.0067031.s017.pdf]

Method S4. Reamplification of primary MSE-PCR products according to Geigl *et al.* 2007 [42]

Reagents:

- Buffer 2 (Roche, 11681 842001)
- dNTP Set,10 mM (New England Biolabs, N0447)
- *Taq*Polymerase (5 U/µl) + 50 mM $MgCl_2$ (Invitrogen, 18038-026)
- LIB1 (5´-AGTGGGATTCCTGCTGTCAGT-3´)

- Mastermix:
  5.0 µl Buffer 2
  2.0 µl 5 mM dNTPs
  2.25 µl $MgCl_2$
  2.0 µl LIB1
  0.5 µl *Taq*Polymerase

- Add 2 µl primary PCR product to the mastermix
- Thermal Cycler program:

95°C	10 min	
95°C	30 sec	45 x
50°C	30 sec	45 x
72°C	2 min	45 x
72°C	7 min	

- Store PCR products at -20°C
