# Supplementary material for: A Robust Method to Analyze Copy Number Alterations of Less than 100 kb in Single Cells Using Oligonucleotide Array CGH
Source: PLoS One. 2013 Jun 25;8(6):e67031. doi: 10.1371/journal.pone.0067031 (PMC3692546; doi:10.1371/journal.pone.0067031)
Supplement: Method S5 — Whole genome amplification of single cell DNA using the Single Cell WGA Kit (WGAN, New England Biolabs, E2620S/L). (PDF) [file pone.0067031.s018.pdf]

Method S5. Whole genome amplification of single cell DNA using the Single Cell WGA Kit (WGAN, New England Biolabs, E2620S/L)

Isolation of single cells :

- Coat the bottom of a petri dish with FCS, dilute cell suspensions with 1x PBS to achieve a density of one cell per visual field under an inverse microscope with 100x magnification
- Use 1 µl pipette to isolate a single cell
- Transfer single cell into 4 µl Cell Extraction Buffer

Whole genome amplification:

- Add 5 µl Extraction Cocktail to each cell and incubate for 10 min at 75°C and 4 min at 95°C in a thermal cycler
- Transfer cells on ice and add 5 µl Pre-Amp Cocktail to each cell
- Preamplification is performed with the following thermal cycler program:

  95°C for 2 min
  95°C for 15 sec
  15°C for 50 sec
  25°C for 40 sec
  35°C for 30 sec } 12 x
  65°C for 40 sec
  75°C for 40 sec

- Place samples on ice
- Add 60 µl Amplification Cocktail to the 15 µl preamplification product
- Amplification is performed with the following thermal cycler program:

  95°C for 2 min
  95°C for 15 sec
  65°C for 1 min } 14 x
  75°C for 1 min

- Store PCR products at -20°C
