# Supplementary material for: A Robust Method to Analyze Copy Number Alterations of Less than 100 kb in Single Cells Using Oligonucleotide Array CGH
Source: PLoS One. 2013 Jun 25;8(6):e67031. doi: 10.1371/journal.pone.0067031 (PMC3692546; doi:10.1371/journal.pone.0067031)
Supplement: Method S6 — Whole genome amplification of single cell DNA using Single Cell WGA Kit (WGAS, Sigma-Aldrich®, WGA4). (PDF) [file pone.0067031.s019.pdf]

Method S6. Whole genome amplification of single cell DNA using Single Cell WGA Kit (WGAS, Sigma-Aldrich®, WGA4)

Isolation of single cells and Proteinase K digestion :

- Coat the bottom of a petri dish with FCS, dilute cell suspensions with 1x PBS to achieve a density of one cell per visual field under an inverse microscope with 100x magnification
- Use 1 µl pipette to isolate a single cell
- Transfer single cell into 8 µl dH_2O
- Add 1 µl Proteinase K Solution in Single Cell Lysis & Fragmentation Buffer to each single cell
- Incubate samples for 1 h at 50°C and 99°C for 4 min
- Place samples on ice

Whole genome amplification:

- Add 2 µl 1x Single Cell Library Preparation Buffer
- Add 1 µl Library Stabilization Buffer
- Incubate samples for 2 min at 95°C in a thermal cycler.
- Cool samples on ice and add 1 µl Library Preparation Enzyme to each cell
- Preamplification is performed with the following thermal cycler program:

  16°C for 20 min
  24°C for 20 min
  37°C for 20 min
  75°C for 5 min

- Add 61 µl Amplification Master Mix (10x Amplification Master Mix, Nuclease-Free Water and WGA DNA Polymerase) to each sample
- Amplification is performed with the following thermal cycler program:

  95°C for 3 min
  94°C for 30 sec } 25 x
  65°C for 5 min }

- Store amplified DNA at -20°C
