# Supplementary material for: A Robust Method to Analyze Copy Number Alterations of Less than 100 kb in Single Cells Using Oligonucleotide Array CGH
Source: PLoS One. 2013 Jun 25;8(6):e67031. doi: 10.1371/journal.pone.0067031 (PMC3692546; doi:10.1371/journal.pone.0067031)
Supplement: Method S7 — Comparison of labeling techniques in aCGH analyses. (PDF) [file pone.0067031.s020.pdf]

Method S7. Comparison of labeling techniques in aCGH analyses

For comparison of labeling methods the amplified DNA was labeled with five different techniques. Random-primed labeling (RP) was performed with the Genomic DNA Enzymatic Labeling Kit (Agilent Technologies) according to the manufacturer`s protocol (Version 6.4). For chemical labeling of the amplified DNA the Universal Linkage System™ (ULS) Labeling Kit (Agilent) was used and the reaction was performed in line with the manufacturer`s instructions. The MSE-PCR based methods were carried out as described by Stoecklein *et al.* [41]. Here the biotin- and digoxigenin-dUTPs were exchanged for directly labeled Cy5-/Cy3-dUTP (MSE1). For the second MSE-PCR (MSE2) based labeling we additionally incorporated Cy5-/Cy3-dCTP in the reaction depicted above. In the fifth PCR-based method Thermo Sequenase (TS) was used for fluorescent labeling with Cy5-/Cy3-dUTP and -dCTP instead of indirectly labeled nucleotides as reported by Fuhrmann *et al.* [20].
