# Supplementary material for: A Robust Method to Analyze Copy Number Alterations of Less than 100 kb in Single Cells Using Oligonucleotide Array CGH
Source: PLoS One. 2013 Jun 25;8(6):e67031. doi: 10.1371/journal.pone.0067031 (PMC3692546; doi:10.1371/journal.pone.0067031)
Supplement: Method S8 — CellSearch® System identification, MoFlo™ XDP isolation and WGAM amplification of single tumor cells. (PDF) [file pone.0067031.s021.pdf]

Method S8. CellSearch® System identification, MoFlo™ XDP isolation and WGAM amplification of single tumor cells

For the preparation of a surrogate CTC sample we spiked cells from the $EpCAM^+$ cell line OE19 in 7.5 ml peripheral blood from a healthy donor. The sample was retained at room temperature for 72 h in a CellSave Preservative Tube (Veridex LLC.). The spiked EpCAM-positive OE19 cells were captured, identified and enumerated using the CellSearch® Epithelial Cell Kit on the CellSearch® System (Veridex LLC.) according to the manufacturer`s protocol. The whole content of the CellSearch® cartridge was transferred into a FACS tube and single cells were sorted into a 96-well plate using fluorescence activated cell sorting (FACS) with the MoFlo™ XDP cell sorter (Beckman Coulter). Each well contained 5 µl of Proteinase K digestion mix (0.5 µl OPA, 0.13 µl 10% Tween, 0.13 µl 10% Igepal, 0.26 µl 10 mg/ml Proteinase K and 3.8 µl dH_2O). Digestion was performed for 10 h at 42°C and Proteinase K was inactivated at 80°C for 10 min.

2 µl MseI-digestion master mix (1 µl MseI, 1 µl OPA) were added to each cell and digestion was performed for 3 h at 37°C. Enzyme was inactivated at 65°C for 5 min. Preannealing of adapters, ligation and primary PCR was performed as described in Method S3. For aCGH hybridization we randomly chosen two single cells of 81 (86.2%) successfully amplified single cells.
