# Supplementary material for: A Robust Method to Analyze Copy Number Alterations of Less than 100 kb in Single Cells Using Oligonucleotide Array CGH
Source: PLoS One. 2013 Jun 25;8(6):e67031. doi: 10.1371/journal.pone.0067031 (PMC3692546; doi:10.1371/journal.pone.0067031)
Supplement: Method S9 — Qualitative and quantitative control of whole genome amplified DNA. (PDF) [file pone.0067031.s022.pdf]

Method S9. Qualitative and quantitative control of whole genome amplified DNA

Quality control of the WGA-DNA was performed by amplification of specific loci in a multiplex-PCR reaction as basically described by Knijnenburg *et al.* [29], (Supplementary Table S1). Essentially six randomly chosen loci from six different chromosomes were checked for successful amplification to gain information of the overall representation of the WGA products. A mastermix was prepared consisting of 5 µl Dream Taq™ Green PCR Master Mix (2X, Fermentas), 1 µl 10X Primer Mix (Supplementary Table S1) and 3.7 µl dH_2O. 0.3 µl template DNA were added to each reaction and reaction was performed as follows: 95°C for 2 min. 95°C for 30 sec, 60°C for 40 sec, 72°C for 1 min (33 cycles). 72°C for 5 min. PCR products were analyzed on a 1.5% agarose gel.
The concentration and yield of the amplified DNA was determined using the Infinite® 200 PRO NanoQuant spectrometer after cleanup of the WGA products with Amicon Ultra-0.5, Ultracel-30 Membrane purification columns (Merck Millipore).
